# Changes in Adipokines following Laparoscopic Roux-en-Y Gastric Bypass Surgery in Chinese Individuals with Type 2 Diabetes Mellitus and BMI of 22–30 kg*·*m^−2^

**DOI:** 10.1155/2013/240971

**Published:** 2013-04-17

**Authors:** Chandrama Shrestha, Honghui He, Yiqun Liu, Shaihong Zhu, Jing Xiong, Zhaohui Mo

**Affiliations:** ^1^Department of Endocrinology and Metabolism, The Third Xiangya Hospital, Central South University, Changsha, Hunan 410013, China; ^2^Department of General Surgery, The Third Xiangya Hospital, Central South University, Changsha, Hunan 410013, China

## Abstract

*Aims.* Although altered endocrine changes following bariatric surgery in morbidly obese patients with diabetes have been demonstrated by previous studies, little is known about their effects on low BMI patients of T2DM. We investigated the changes in adipokines and sICAM-1 in Chinese subjects with low BMI and T2DM after LRYGB and explored their relationship with postsurgical insulin sensitivity. *Methods.* Plasma levels of adiponectin, sICAM-1, fasting glucose, glycated hemoglobin, and fasting insulin and serum levels of visfatin were measured before and at three months after LRYGB in 33 T2DM patients with BMI of 22–30 kg*·*m^−2^. *Results.* Significant reductions in anthropometric measurements and indicators of glucose and lipid metabolism and moderate reductions in insulin resistance and fasting insulin were observed at three months after LRYGB. Postoperative adiponectin level (*P* < 0.001) was increased compared to the preoperative level, whereas visfatin (*P* < 0.001) and sICAM-1 (*P* < 0.001) were lower than that before surgery. Serum adiponectin negatively correlated with HOMA-IR and FIns both preoperatively and at three months after surgery, and visfatin positively correlated with HOMA-IR and FIns both preoperatively and postoperatively. *Conclusion.* Changes in adipokines were related to an improvement in postsurgical insulin sensitivity, which was predicted by weight loss after LRYGB even in low BMI patients with T2DM.

## 1. Introduction

Diabetes mellitus has become a public health problem for the past couple of decades, and the exploration for therapies to combat this continues unabated. Bariatric surgery is already recognized as an effective therapeutic option for producing weight loss in morbidly obese patients, and it not only leads to subsequent weight loss but also ameliorates or resolves various comorbidities including type 2 diabetes mellitus [1], and vast research has been undergone regarding the mechanism underlying this in an attempt to understand it hoping that it would pave a way for new and more efficient therapeutic strategies. It has also been considered beneficial in nonobese patients with diabetes [2], but scant literature is available regarding low BMI patients with diabetes in context to bariatric surgery [2–4]. Adiponectin is an idiosyncratic collagen-like cytokine secreted by mature fat cells, and it directly regulates glucose metabolism and insulin sensitivity in vitro and in vivo through the activation of AMP-activated protein kinase (AMPK) [5]. It also increases fatty acid oxidation in skeletal muscle cells by sequential activation of AMP-activated protein kinase, p38 mitogen-activated protein kinase, and peroxisome proliferator-activated receptor-alpha, thereby contributing to enhanced insulin sensitivity [6]. Visfatin exerts insulin-mimetic effects in cultured cells and lowers plasma glucose levels in mice, and it binds to and activates the insulin receptor [7]. Visfatin has a role in inhibiting apoptosis of *β*-cells through the activation of MAPK-dependent and phosphoinositide 3-kinase (PI3K)-dependent signaling pathway [8]. Visfatin is involved in inflammatory process, and sICAM-1 plays an important role in diabetic vascular disease. So, we investigated the endocrine and metabolic changes post-LRYGB in Chinese patients with T2DM, whose body mass indices were between 22 and 30 kg·m^−2^, focusing on adiponectin, visfatin, and sICAM-1 and explored their relationship with postsurgical insulin sensitivity.

## 2. Materials and Methods

### 2.1. Patients

Thirty-three patients of T2DM (BMI 26.71 ± 0.69 kg·m^−2^) (aged 49.51 ± 1.33 years), who underwent LRYGB performed in The Third Xiangya Hospital of Central South University from June 2009 to July 2011, were randomly included in this case study. Among them, twenty-four were male, and nine were females. Inclusion criteria included (1) diagnosis of T2DM based on the guidelines set by the ADA in 2007, (2) the ratio of peak to the basic value of CPRT > 2 (the basic value of CPRT by OGTT > 0.33 *μ*g/L), (3) BMI: 22–30 kg·m^−2^, (4) age ≤ 65 years, and (5) duration of diabetes < 10 years. The inclusion of patients was based on all criteria simultaneously. Exclusion criteria included patients with a history of open abdominal surgery, unstable psychiatric illness, inability or reluctance to cooperate in follow-up, alcohol or drug dependence, and relatively high surgical risks such as active ulcer or *Helicobacter pylori* infection. Fasting blood samples were collected prior to the surgery and at 3 months after surgery. The study was conducted in compliance with the protocol approved by the Ethics Committee of The Third Xiangya Hospital, Central South University, and all patients provided written informed consents for participation in the study.

### 2.2. Surgical Procedure

All the operations were performed in the Department of Surgery of The Third Xiangya Hospital of Central South University. The proximal jejunum was transected 40 cm distal to the ligament of Treitz, the alimentary limb was measured at 100 cm, and a side-to-side jejunostomy was performed using 60 mm linear cutter and 3.5 mm stapler. The jejunostomy and the mesenteric defects were subsequently closed with manually placed polyglactin (2–0 Vicryl, Ethicon, USA) sutures, and with nylon (2–0 Ethibond, Ethicon, UK) sutures respectively. A 25 ml proximal gastric pouch was created with 60 mm linear cutter and 3.5 mm stapler. Then, a 2.5 cm long gastrojejunostomy was formed with 30 mm linear cutter and 2.5 mm stapler at the antecolic position. The gastrojejunostomy was closed with manual sutures (2–0 Vicryl, Ethicon, USA), and a drain was placed near gastrojejunostomy around the right-upper quadrant.

### 2.3. Anthropometric and Biochemical Measurements

Standard methods were used to measure waist and hip circumferences, height, and weight. The biochemical measurement for visfatin was performed on fasting serum samples. All other biochemical measurements were performed on fasting plasma samples. Plasma glucose was measured with glucose analyzer (EKF BIOSEN C-line glucose lactate analyzer, Germany) and glycated hemoglobin by high performance liquid chromatography. Insulin was measured by chemiluminescence (Advia Centaur XP, Siemens, Germany). Homeostatic model assessment of insulin resistance was calculated as a product of fasting plasma glucose and insulin divided by a constant (HOMA-IR = (FPG (mmol/L) × FIns)/22.5) [9]. Adiponectin, sICAM-1, and visfatin were measured by ELISA (RD Company, USA).

### 2.4. Statistical Analysis

Statistical analysis was done using SPSS 13.0. Data are expressed as mean ± SD. Comparisons were made using the *t*-test for data following the normal distribution or rank sum test for data not following the normal distribution. Analysis of the preoperative and postoperative differences was done using the Pearson correlation for data following the normal distribution or the Spearman analysis for data not following the normal distribution. Multivariate analysis was done by multiple regression analysis. The level of significance is taken at *P* < 0.05. 

## 3. Results

### 3.1. General Outcome (Table 1)

Three-month postoperatively, statistically significant decrease in both BMI (*P* < 0.001) and WHR (*P* < 0.01) was noted.

### 3.2. Indicators of Glucose and Lipid Metabolism (Table 1)

Three months after RYGB, a significant decrease in the levels of FPG, 2HPG, HbA1c, and TG was noted. HOMA-IR and FIns were also reduced but were not statistically significant. 

### 3.3. Serum Adiponectin, Visfatin, and sICAM-1 Levels (Table 2)

**Figure 1:**
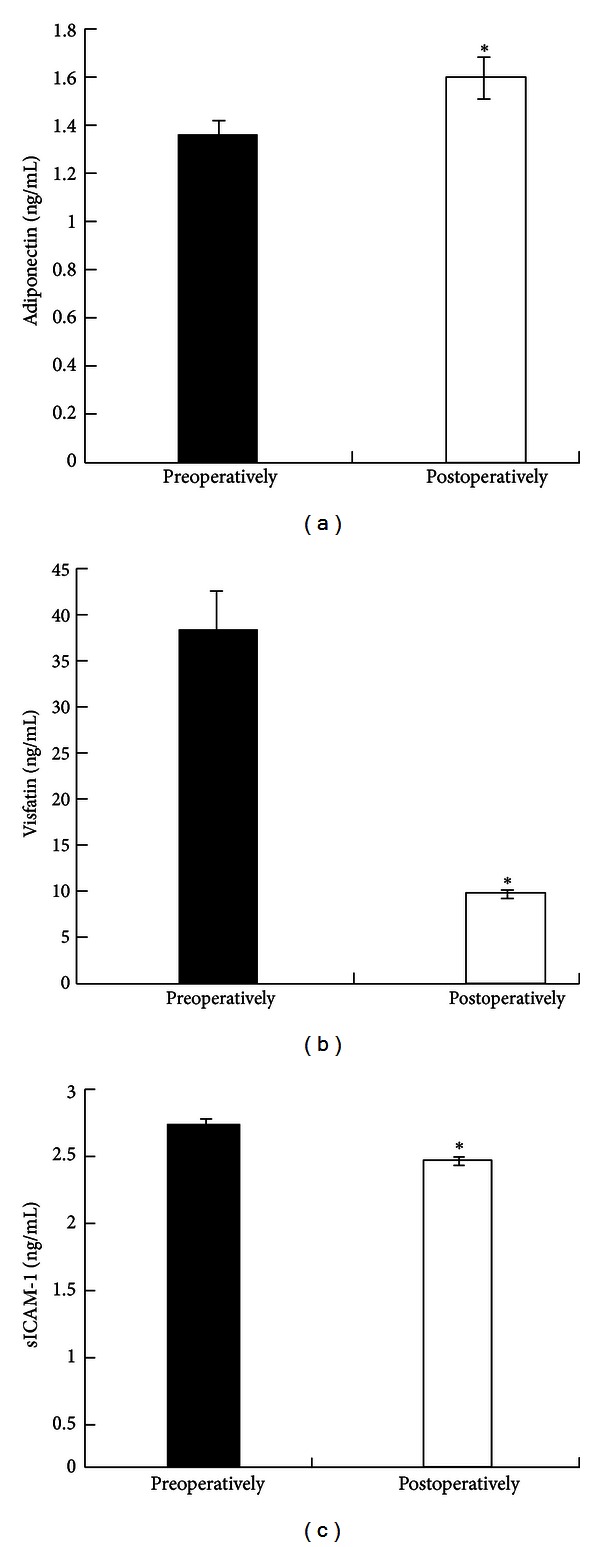
Fasting plasma concentration of (a) Adiponectin, (b) Visfatin, (c) sICAM-1 preoperatively and at 3 months after surgery.

Three-month postoperatively, a statistically significant increase (*P* < 0.001) in the concentration of adiponectin and significant reductions (*P* < 0.001) in the concentration of visfatin and sICAM-1 were observed (Figure 1).

Concentrations of adipocyte hormones were further correlated with HOMA-IR and FIns. Both at baseline and at 3 months after surgery, adiponectin negatively correlated with HOMA-IR and Fins, whereas plasma visfatin positively correlated with HOMA-IR and FIns. A significant difference was found between the mean BMI of postoperative participants whose adiponectin values were below and above the median of adiponectin [*t*(29) = 2.39, *P* < 0.05]. The mean BMI of the postoperative participants whose adiponectin levels were below the median was significantly higher (25.81 ± 4.08 kg·m^−2^) than of those whose adiponectin levels were above the median (22.96 ± 2.38 kg·m^−2^). However, there was no significant correlation of these adipocyte hormones with BMI, WHR, FPG, 2HPG, HbA1c, TG, CHOL, HDL-CH, LDL-C, or sICAM-1. Serum sICAM-1 levels and changes in BMI, WHR, FPG, 2HPG, HbA1C, TG, CHOL, HDL-CH, LDL-C, HOMA-IR, FIns, adiponectin, and visfatin were not correlated.

## 4. Discussion

Global cutoff points for overweight and obesity as set by the World Health Organization are BMI of 25.0 kg·m^−2^ and 30.0 kg·m^−2^, respectively [10]. There has been a lot of controversy regarding these cutoff points in Asian populations for more than a decade. Few studies in China [11, 12], Japan [13], Taiwan [14], and Hong Kong [15] have reported an association between a BMI of 22.3 kg·m^−2^ and increased atherogenic risk factors. A survey by Kim et al. reported that the prevalence of diabetes, hypertension, and dyslipidemia has doubled at a BMI of 23.0 to 24.0 kg·m^−2^ and tripled at a BMI of 26.0 kg·m^−2^ in the Korean adult population and that body fat and fat distribution at a BMI of 23.0 kg·m^−2^ in Asians may be similar to those in the Whites at a BMI of 25.0 kg·m^−2^ [16]. The WHO, the International Obesity Task Force, and the International Association for the Study of Obesity have proposed lower cutoff points for overweight (BMI 23.0 kg·m^−2^) and obesity (BMI 25.0 kg·m^−2^) in Asian and Pacific Island populations [17]. A study by Huang et al. has showed that Roux-en-Y gastric bypass could be used in Chinese T2DM patients with BMI of 25–35 kg·m^−2^, and 63.6% patients showed T2DM remission, 27.3% showed glycemic control, and 9.1% showed improvement at 12-month duration in their study [18]. The BMI cutoff for overweight in our study was 23 kg·m^−2^ and that for obesity was 28 kg·m^−2^. In our study, 20 patients were overweight and 9 patients were obese. Some patients had raised triglyceride, cholesterol and/or LDL levels, and/or microalbuminuria. 

Our study investigated the changes of adipocyte hormones and systemic marker of inflammation following LRYGB in low BMI Chinese subjects with T2DM. The mean BMI in our study was (26.71 ± 0.69 kg·m^−2^), and the mean age of the patients was (49.51 ± 1.33 years). The HbA1c level of all patients exceeded 7% despite being on maximum tolerated dose for a duration of at least six months. The major limitations of our study were that the sample size was limited to 33 individuals only, and as the sample was randomly selected, the patients could not be equally weighted for sex to allow for between sex differences. Expanding the sample size and the duration of study would further validate the results.

While previous studies have reported the endocrine and metabolic changes post-RYGB in obese patients with T2DM, we believe this to be the first study to report these findings in case of low BMI patients with T2DM. The patients were on oral hypoglycemics or insulin or both prior to the surgery. Among the 33 patients, 19 (45.4%) were on oral hypoglycemic agents prior to surgery, and 8 (21.2%) were on insulin. The other 6 (33.3%) were using both oral hypoglycemic and insulin. After the surgery, 8 (24.2%) patients totally came off these medications, whereas 4 (12.1%) remained on insulin. The other 17 (51.5%) were on oral hypoglycemic agents but on a lesser dose than that before surgery.

Three months after the surgery, statistically significant amelioration in body mass index and waist-hip circumference could be observed in our study. In addition, the indicators of glucose metabolism, namely, HbA1c, FPG, and 2HPG were significantly reduced in comparison to the preoperative values. Likewise, statistically significant reduction in TG could be seen. At three months post-LRYGB, decrease in HOMA-IR and FIns levels was also noted, suggesting that LRYGB could be beneficial even for low BMI individuals of T2DM (Table 1). Similar findings have been reported by several studies conducted on obese individuals with diabetes [19–23]. 

The results of our study reveal that there was an increase in the adiponectin levels (*P* < 0.001) three months after LRYGB (Table 2). Few other studies have come up with similar results albeit on different time frames after-surgery (at 6 and 12 months) and on morbidly obese subjects [24–26].

Three-month postoperatively, a statistically significant reduction in the concentration of visfatin (*P* < 0.001) was noted (Table 2). There has been conflicting results regarding the study of visfatin post-RYGB. Some studies have reported decreased levels of visfatin post-RYGB [23, 27] in consistent with the findings of our study, whereas a study reported an increase [28]. The results for disparate visfatin levels are unknown but may be attributed to the variation in the gastrointestinal surgery, the study population, the duration of postoperative observation, the degree of inflammatory status, and the surgical approach.

Our study found that the serum adiponectin negatively correlated with HOMA-IR and FIns both preoperatively and at 3 months after surgery, and visfatin positively correlated with HOMA-IR and FIns both preoperatively and postoperatively (Table 3). In addition, multivariate analysis revealed that the changes in insulin sensitivity were predicted by weight loss. No significant correlation of these adipocyte hormones with BMI, WHR, FPG, 2HPG, HbA1c, TG, CHOL, HDL-CH, LDL-C, or sICAM-1 was found. Varied results regarding this have been found by other investigators though. Swarbrick et al. reported that the proportional improvement in HOMA-IR over 12 months in their study was related to change in adiponectin [23]. Another prospective study on 31 morbidly obese patients followed up for a duration of 6 months showed that insulin sensitivity as estimated by the HOMA-IR was unchanged, but individual changes of insulin resistance and visfatin were significantly associated [29]. 

RYGB in individuals with T2DM not only leads to reductions in blood sugar, blood lipids, blood pressure, and other metabolic disorders but also improves the inflammatory status of patients with T2DM [30]. Our study shows that the level of sICAM-1 was lower at 3-month post-LRYGB than that before surgery (*P* < 0.001) (Table 2). Other studies reported similar results on morbidly obese subjects at 3, 6, and 12 months [23, 31]. 

## 5. Conclusion 

Changes in adipokines are related to an improvement in postsurgical insulin sensitivity even in low BMI patients with T2DM, and the major predictor was weight loss. Unraveling the pathogenesis and attaining in-depth exploration would require large-scale clinical studies and long-term followup that would further elucidate the effect of LRYGB in such patients.

## Figures and Tables

**Table 1:** Comparison of indicators of glucose and lipid metabolism in patients with T2DM preoperatively and at 3 months ($\overset{-}{x}\pm s$).

	Preoperatively	3-month postoperatively
*N*	33	33
BMI (kg · m^−2^)	26.71 ± 0.69	24.53 ± 0.62***
WHR	0.96 ± 0.01	0.92 ± 0.01**
HbA1c (%)	8.01 ± 0.29	6.79 ± 0.16***
FPG (mmol/L)	8.94 ± 0.35	7.10 ± 0.32***
2HPG (mmol/L)	12.76 ± 0.67	9.29 ± 0.39***
FIns (uIU/mL)	11.57 ± 2.70	9.11 ± 2.26
HOMA-IR	3.77 ± 0.89	2.71 ± 0.76
TG (mmol/L)	2.26 ± 0.47	1.39 ± 0.12**
CHOL (mmol/L)	4.46 ± 0.21	4.24 ± 0.14

**P < 0.01 and ***P < 0.001; comparison of parameters before surgery and 3 months after surgery.

**Table 2:** Comparison of adipokines and sICAM-1 preoperatively and 3-month postoperatively.

	Preoperatively	3-month postoperatively
*N*	33	33
Adiponectin (ng/mL)	1.36 ± 0.07	1.60 ± 0.09***
Visfatin (ng/mL)	38.24 ± 5.32	9.79 ± 0.64***
sICAM-1 (ng/mL)	2.70 ± 0.06	2.42 ± 0.03***

**P < 0.01 and ***P < 0.001; comparison of parameters before surgery and 3 months after surgery.

**Table 3:** Correlation matrix of adipokines with HOMA-IR and FIns.

	HOMA-IR		FIns	
	Preoperatively	3-month postoperatively	Preoperatively	3-month postoperatively
Adiponectin	−0.722***	−0.482**	−0.713***	−0.505**
Visfatin	0.678***	0.449*	0.675***	0.423*

*P < 0.05, **P < 0.01, and ***P < 0.001.

## Abbreviations

ADA: American diabetes association
BMI: Body mass index
CHOL: Cholesterol
CPRT: C-peptide release test
DM: Diabetes mellitus
ELISA: Enzyme-linked immunosorbent assay
Fins: Fasting insulin
FPG: Fasting plasma glucose
HbA1c: Glycosylated hemoglobin
HOMA-IR: Homeostatic model assessment-insulin resistance
LRYGB: Laparoscopic Roux-en-Y gastric bypass
OHA: Oral hypoglycemic agents
OGTT: Oral glucose tolerance test
RYGB: Roux-en-Y gastric bypass
sICAM-1: Soluble intracellular adhesion molecule-1
TG: Triglyceride
WHR: Waist-hip ratio
T2DM: Type 2 diabetes mellitus
2HPG: 2-hour postprandial plasma glucose.
